# Telemedicine-assisted structured self-monitoring of blood glucose in management of T2DM results of a randomized clinical trial

**DOI:** 10.1186/s12911-023-02283-4

**Published:** 2023-09-14

**Authors:** Chen-Yu Han, Jian Zhang, Xiao-Mei Ye, Jia-Ping Lu, Hai-Ying Jin, Wei-Wei Xu, Ping Wang, Min Zhang

**Affiliations:** 1 Department of Endocrinology, Qingpu Branch of Zhongshan Hospital affiliated to Fudan University, No.1158 of Gongyuan Road, Qingpu District, Shanghai, 201700 China; 2 Department of Pharmacology, Medical College of Shaoxing University, Shaoxing, 312000 China

**Keywords:** Type 2 diabetes mellitus, Self-monitoring, Telemedicine, Self-management, Glycemic control

## Abstract

**Background:**

This prospective study aimed to compare telemedicine-assisted structured self-monitoring of blood glucose(SMBG) with a traditional blood glucose meter (BGM) in adults of type 2 diabetes mellitus (T2DM).

**Methods:**

Adult participants with T2DM were assigned to an intervention group or a control group. The patients in the intervention group received a connected BGM with real-time data submission as well as individual needs-based tele-coaching to address and improve motivation and daily diabetes self-management. The patients in the control group received a traditional BGM. Changes in glycated hemoglobin(HbA1c), low blood glucose index(LBGI), and diabetes self-management behaviors were analyzed.

**Results:**

The study demonstrated the superiority of the telemedicine-assisted structured SMBG versus the traditional BGM for improving HbA1c. Additionally, the telemedicine-assisted SMBG reduced the risk of hypoglycemia and enhanced diabetes self-management behaviors, as differences in the LBGI and the Diabetes Self-Management Questionnaire(DSMQ) results between the groups after 6 months were found to be significant.

**Conclusions:**

Telemedicine-assisted structured SMBG helps physicians and patients to achieve a specific level of glycemic control and reduce hypoglycemia. The use of coaching applications and telemedicine-assisted SMBG indicated beneficial effects for T2DM self-management, which may help limit disease progression.

**Trial registration:**

Chinese Clinical Trail Registry No: ChiCTR2300072356 on 12/06/2023. Retrospectively registered.

## Introduction

Diabetes mellitus (DM) is one of the world’s most serious non-infectious diseases and a major threat to human health. The World Health Organization estimates that 366 million patients will be suffering from diabetes by 2030, twice the number of patients in 2000 [1].

DM is classified into type 1 DM (T1DM) and type 2 DM (T2DM), with T2DM accounting for nearly 95% [2]. T2DM is characterized by insulin resistance and insulin secretion deficiency [3], which can cause multiple organ injuries and numerous complications [4]. The complications of T2DM not only result in serious damage to physical and mental health, ultimately reducing life span, but they also place economic burdens on both individuals and society.

The incidence and severity of complications mainly depend on the course of diabetes and the control of blood glucose. Therefore, good metabolic control is very important for patients with T2DM. In China, control of blood glucose (BG) and other metabolic indicators for patients with T2DM mainly depends on doctors, which is relatively standardized. However, the management of diabetic patients outside hospitals is chaotic, lacking homogeneity, and mainly depends on the educational level and participation of patients. Therefore, the self-management of diabetic patients and appropriate management strategies are particularly important [5]. Despite the evidence that the self-monitoring of BG (SMBG) and a healthy lifestyle are beneficial for controlling the disease, the implementation of effective SMBG and long-term lifestyle changes is a huge daily challenge [6]. The disadvantages of SMBG are related mainly to individual patients, who may lack the motivation for testing or may not be sufficiently aware of how/when to test and how to interpret the results [7].

Technology has long been used for self-management and to improve treatment compliance in people with diabetes [8–10]. Systems based on telephone coaching, text message service support, or telemedicine have proven effective in increasing management compliance, and consequently, improving glycemic control [9, 10]. The global implementation of mobile phones has fostered the development of applications (app) for managing diabetes, which have become primary tools for decision support and disease management for both people with diabetes and healthcare providers [11]. The newly developed telemedicine system is a multifunctional combination of monitoring, counseling, and lifestyle intervention, and it enables the personalization of SMBG. Compared with the conventional methods of chronic disease management, the telemedicine system not only emphasizes the importance of patients’ self-management but also establishes appropriate communication between patients and healthcare providers, thereby contributing to a reduction in hospital visits [12–14].

Telemedicine is a very promising tool for delivering personalized SMBG and healthcare at home or where it is needed, reducing the unnecessary use of healthcare resources [15, 16]. However, there remains a lack of consistent information on concrete clinical outcome measures of specific illnesses, such as changes in glycated hemoglobin (HbA_1c_) levels in diabetes [17]. One drawback is the lack of knowledge regarding the effectiveness of telemedical devices in promoting diabetes management. Evidence of such efficacy might impact the overall burden of the disease by reducing the unnecessary use of healthcare resources [18]. Thus, based on the premise that telemedicine-assisted structured SMBG may increase the efficacy of treatment and self-regulation, this study aimed to evaluate the effects of telemedicine-assisted structured self-monitoring of blood glucose on glycemic control and diabetes management of Chinese patients.

## Research design and methods

### Participant recruitment

This study was an open-label randomized (1:1) trial involving patients with T2DM with suboptimal glycemic control (7% ≤ HbA1c ≤ 11%), aged 18–75 years, with a diabetes duration of 5–10 years. The exclusion criteria were acute infection, malignant tumor, liver failure, pregnancy or lactation, long-term cortisol treatment, and inability to use smartphone applications. Participants were randomized by Qingpu Branch of Zhongshan Hospital affiliated to Fudan University, Shanghai, China from June 2021 to June 2022. All participants provided signed informed consent before enrolment in the study. And the study was registered at http://www.chictr.org.cn/index.aspx (No: ChiCTR2300072356) on 12/06/2023.

### Intervention

**Fig. 1:**
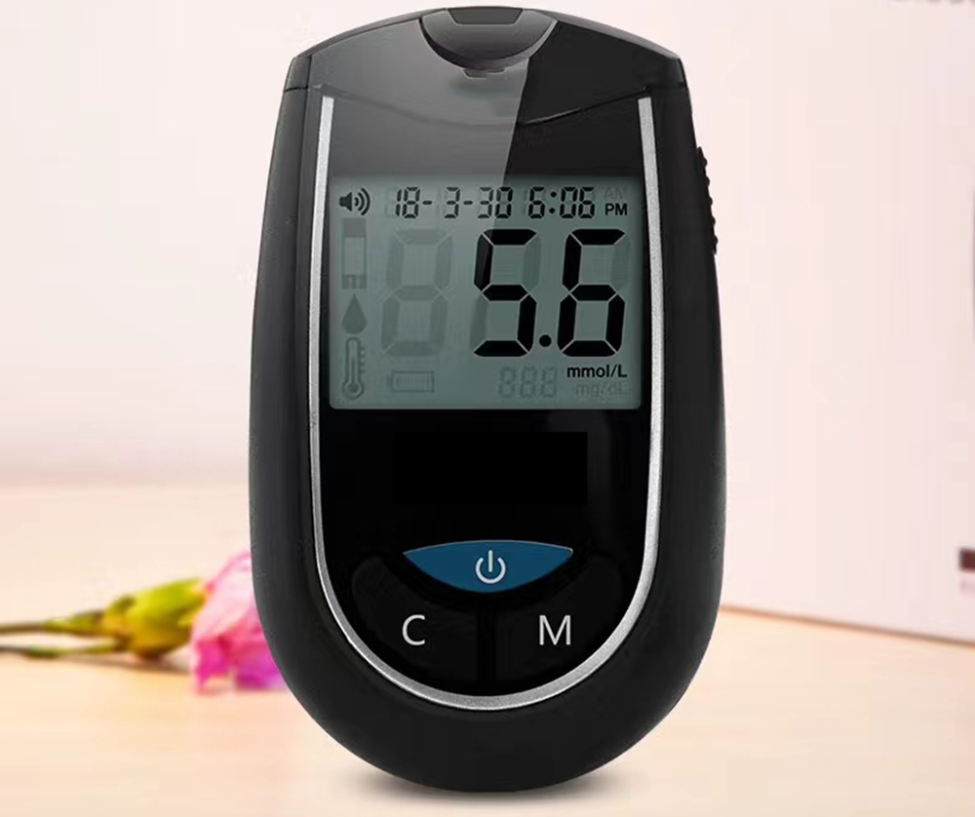
The blood glucose meter named SINOMEDISITE.

The participants were allocated randomly to an intervention group or a control group. The study was not blinded. Before the study, the patients in both groups received a self-management guide and a blood glucose meter(BGM). The name of the BGM was SINOMEDISITE as showing in Fig. 1. For the patients in both groups, the following monitoring strategy was devised: The structured monitoring consisted of six points (before each meal and 2 h after eating) if three main meals were consumed daily and whenever there was a risk of hypoglycemia, especially at night (i.e., the time of the highest risk of hypoglycemia). However, only the BGM given to the patients in the intervention group was connected to the telemedicine system through Bluetooth, and the SMBG data were transmitted to the telemedicine system in real time. The data were displayed as in-range (green), high (yellow), and low (red) BG levels, and a curve was created. Both patients and physicians could see these values via the mobile app (Huayi Glucose Butler). The telemedical device served as a feedback tool for the patients to provide insights into the impact of behavioral changes on their BG levels. Moreover, patients in the intervention group received beneficial advice from the app, including an introduction to diabetes, diet and fitness programs, and overall lifestyle adjustments [19]. The app also enabled secure data transfer and communication between patients and physicians; the submitted data was interpreted, and personal health goals were developed to help patients better control the disease step by step [20].

Compared with the intervention group, the subjects in the control group could only examine and save their physiological parameters, which were eventually delivered to physicians for further medical advice. All the subjects had weekly phone-based coaching sessions in which they were encouraged to use the devices to actively control and manage their diabetes as part of their daily lives. The patients in both groups received routine care for medicine adjustment and condition assessment and visit hospital once a month.

### Data collection and measures

The program ran for a period of 6 months. After 3 months, the results were collected and analyzed to address the program’s efficacy. The primary objective of this study was to demonstrate superiority of telemedicine-assisted structured monitoring as a component in diabetes management versus traditional self-monitoring in reducing HbA1c levels after 6 months. The secondary efficacy endpoints after 6 months were the additional measurement of low BG index (LBGI) values and self-management behaviors. Other endpoints were the metabolic index (fasting BG [FBG], body mass index [BMI], blood pressure [BP], total cholesterol [TC], triglycerides [TG], high-density lipoprotein [HDL], and low-density lipoprotein [LDL]) and the frequency of outpatient services and hospitalization.

Data were collected from routine laboratory results, the coaching app, and online research questionnaires, as described below. Physiological parameters (including laboratory examination results, BMI, and BP) were measured by physicians at baseline, 3 months, and 6 months. Venous blood was collected after overnight fasting. Laboratory indicators (FBG, 2-h postprandial BG [2hBG], HbA1c, TC, TG, HDL, and LDL) were analyzed in laboratories in the Qingpu branch of Zhongshan Hospital, which is affiliated with Fudan University. Body weight was measured while wearing light clothing, with a deviation of 0.1 kg (weight) and 0.5 cm (height) [21]. Blood pressure was determined by the average value of two measurements, which were taken at an interval of 5 min with the patient in a sitting position. The numbers of outpatient services used and hospitalizations were assessed according to the patient’s medical records.

Diabetes self-management was assessed using the Diabetes Self-Management Questionnaire (DSMQ) [22], which was based on specific diabetes-related questions (see Supplementary Material). The 16-item scale reflected different aspects of diabetes self-management components, including dietary control, medication compliance, BG monitoring, physical activity, and physician contact. The LBGI was used to evaluate the frequency and degree of hypoglycemia in SMBG, based on the mathematical processing of BG measurements. The specific calculation methods were as follows:

1) The BG value was transformed:

$${X}_{i}=1.794\times \left\{{\left[\left(\text{ln}{G}_{i}\right)\right]}^{1.026}-1.861\right\}$$

*Xi* is the converted BG; G is the measured BG.

2) The risk value for BG was calculated according to *Xi*:

$$\text{L}\text{B}\text{G}\text{I}=\frac{1}{N}{\sum }_{t=0}^{N}\text{r}\text{l}\left({\text{x}}_{\text{i}}\right)$$

N is the total number of blood glucose measurements; rl is the risk of hypoglycemia(*Xi < 0*). To calculate the LBGI, the BG was detected at least four times randomly per day for 1 month [23].

### Statistical analysis

Assuming a standard deviation of HbA1c of 0.9% and considering as clinically relevant a minimum between group difference in HbA1c levels of 0.4%, the number of patients to be enrolled to ensure a power of 80% (alpha = 0.05) was 121 patients per arm. Assuming a dropout rate of 40%, 410 patients were needed. Randomization was performed through sealed envelopes. Random lists were computer generated. The baseline characteristics or data after intervention were summarized as mean and standard deviation (SD) (for continuous, normally distributed variables), median and interquartile range (for continuous, not normally distributed variables), or percentage (for categorical variables). The baseline characteristics and 6-month characteristics were compared between arms using an unpaired *t* test or the Chi-squared test. Data before and after intervention in the same group were compared using a paired *t* test or the Chi-squared test. Missing values due to dropout or loss of follow-up were imputed using the mean of each group at 6 months. A two-tailed *P* value *<* 0.05 was considered statistically significant. Data were analyzed using SPSS Statistics 25 software.

## Results

**Fig. 2:**
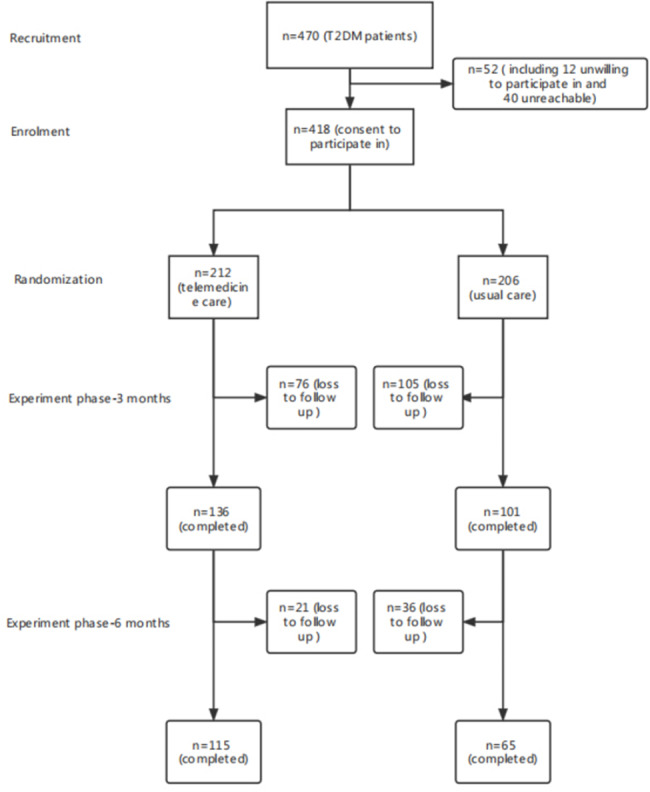
Study design and flow chart

As demonstrated in Fig. 2 and 418 patients were randomized into the intervention group (*n* = 212) or the control group (*n* = 206). Overall, 136 (64%) participants in the intervention group and 101 (49%) participants in the control group completed 3 months of intervention. Finally, 65 (31%) participants completed the 6- month follow-up, as did 115 (54%) intervention participants. The dropout rate in the control group was significantly higher than that in the telemedicine group (*P* = 0.001). The baseline characteristics of the two groups were similar (see Table 1), and they did not show obvious differences between the participants who completed the intervention and those who dropped out. No important harms or unintended effects in both group.

**Table 1:** Patients’ characteristics on baseline

Characteristics	Telemedicine(212)	Control(206)	*p* value*
Age (years)	52.1 ± 9.2	51.8 ± 8.3	0.68
Males (%)	62(45.5)	48(47.5)	0.85
Diabetes duration (years)	7.4 ± 1.5	7.2 ± 1.5	0.46
FBG (mmol/L)	7.1 ± 1.6	7.0 ± 1.6	0.75
2hPBG (mmol/L)	9.7 ± 2.3	9.6 ± 2.2	0.69
HbA1c (%)	7.9 ± 0.9	8.0 ± 0.9	0.56
BMI (Kg/m^2^)	24.6 ± 1.9	24.2 ± 1.5	0.18
Systolic BP (mmHg)	125.4 ± 13.3	125.5 ± 13.9	0.95
Diastolic BP (mmHg)	76.6 ± 9.1	77.17 ± 10.8	0.84
Triglycerides (mmol/L)	2.4 ± 0.8	2.6 ± 1.0	0.40
Total cholesterol (mmol/L)	5.4 ± 0.7	5.5 ± 0.8	0.91
HDL cholesterol (mmol/L)	1.1 ± 0.4	1.1 ± 0.3	0.95
LDL cholesterol (mmol/L)	3.7 ± 0.7	3.8 ± 0.8	0.79
Glucose control methods			
OAD only	97(0.71)	78(0.77)	0.64
Insulin only	2(0.01)	1(0.01)	0.51
OAD + insulin	38(0.28)	22(0.22)	0.48
Educational status			
Elementary	34(25)	26(25.7)	0.55
Middle	73(53.6)	56(55.4)	0.68
High	27(19.8)	18(17.8)	0.29
College	2(1.4)	1(0.9)	0.17
Testing frequency(times/month)	1.9 ± 0.3	1.7 ± 0.6	0.86

Data are means ± SD or n (%). FBG, fasting blood glucose; BMI, body mass index; BP, blood pressure; HDL, high-density lipoprotein; LDL, low-density lipoprotein; OAD, oral hypoglycemic drugs

*P<0.05

### Primary outcome

At 6 months, the unadjusted mean HbA1c values were 7.38% for the intervention group and 7.98% for the control group(*P* < 0.001). The participants in the intervention group had lower HbA1c values than the participants in the control group, as revealed in Table 2. Additionally, the reduction in HbA1c levels is independent of the BGM testing frequency. As shown in Table 2, the BGM test frequency of subjects in telemedicine group decreased in the last 3 months, but the level of HbA1c still decreased. The paired *t* test found a significant difference within the intervention group but not within the control group. The independent-samples *t* test revealed a significant difference between the intervention and control groups after 6 months. A comparison of the percentage of readings in-range (RIR) in 2 weeks showing significant difference within the intervention group and between the two groups(*P* < 0.05), as revealed in Table 2.

**Table 2:** Comparison of the differences between groups

	Telemedicine group				Control group				*P* value	
	Baseline (n = 212)	3 Month (n = 136)	6 Month (n = 115)	*P*	Baseline (n = 206)	3 Month (n = 101)	6 Month (n = 65)	*P*	3 Month	6 Month
FBG (mmol/L)	7.12 ± 1.6	6.81 ± 0.9	6.93 ± 1.2	0.23	7.05 ± 1.6	6.91 ± 0.8	7.13 ± 1.1	0.65	0.45	0.32
HbA1c (%)	7.95 ± 0.9	7.52 ± 0.9	7.38 ± 1.1	0.00*	8.03 ± 0.9	7.77 ± 1.1	7.98 ± 0.1	0.38	0.11	0.00*
LBGI	2.62 ± 1.8		2.12 ± 0.9	0.03*	2.71 ± 1.2		2.52 ± 1.1	0.22		0.04*
DSMQ (sum scale)	6.75 ± 1.8		7.79 ± 1.2	0.00*	6.13 ± 1.9		6.76 ± 1.2	0.58		0.00*
BMI (Kg/m^2^)	24.61 ± 1.9	23.64 ± 1.3	23.17 ± 0.8	0.17	24.22 ± 1.5	24.35 ± 2.2	24.98 ± 0.6	0.44	0.42	0.21
Systolic BP (mmHg)	125.42 ± 13.3	123.25 ± 11.1	124.68 ± 9.8	0.90	125.51 ± 13.9	127.94 ± 12.5	124.17 ± 8.6	0.79	0.21	0.63
Diastolic BP (mmHg)	76.61 ± 9.1	74.94 ± 8.5	75.67 ± 6.3	0.73	77.17 ± 10.8	76.12 ± 12.3	75.8 ± 7.6	0.67	0.35	0.88
Triglycerides (mmol/L)	2.52 ± 0.8	2.15 ± 0.7	1.78 ± 0.4	0.00*	2.61 ± 1.0	2.34 ± 0.9	2.47 ± 1.8	0.82	0.67	0.00*
Total cholesterol (mmol/L)	5.41 ± 0.7	5.24 ± 1.3	5.37 ± 0.8	0.66	5.52 ± 0.9	5.45 ± 1.1	5.38 ± 1.2	0.43	0.73	0.92
HDL cholesterol (mmol/L)	1.13 ± 0.4	1.26 ± 0.3	1.14 ± 0.9	0.99	1.12 ± 0.3	1.08 ± 0.6	0.96 ± 0.6	0.24	0.42	0.11
LDL cholesterol (mmol/L)	3.81 ± 0.7	3.65 ± 0.6	3.59 ± 1.1	0.34	3.73 ± 0.8	3.74 ± 0.9	3.68 ± 1.0	0.87	0.76	0.91
BGM testing frequency(times/month)	1.90 ± 0.3	9.84 ± 0.5	6.97 ± 0.6	0.01*	1.72 ± 0.6	6.58 ± 0.6	3.69 ± 0.6	0.04*	0.03*	0.01*
RIR in two weeks(%)	51	72	68	0.04*	55	61	58	0.68	0.01*	0.02*
Hypoglycemic episodes (%)			6(4.4)				5(4.9)			0.71
Emergency department visits (%)			5(3.6)				3(2.9)			0.86

Data is means ± SD, median and interquartile range, or n (%). FBG, fasting blood glucose; HDL, high-density lipoprotein; LDL, low-density lipoprotein; RIR, readings in-range (70 to 180 mg/dl); RIR in two weeks: RIR in the first 2 weeks of testing and in the 2 weeks prior to the 3 month and in the 2 weeks prior to the 6 month visit

*P<0.05

### Secondary outcomes

**Fig. 3:**
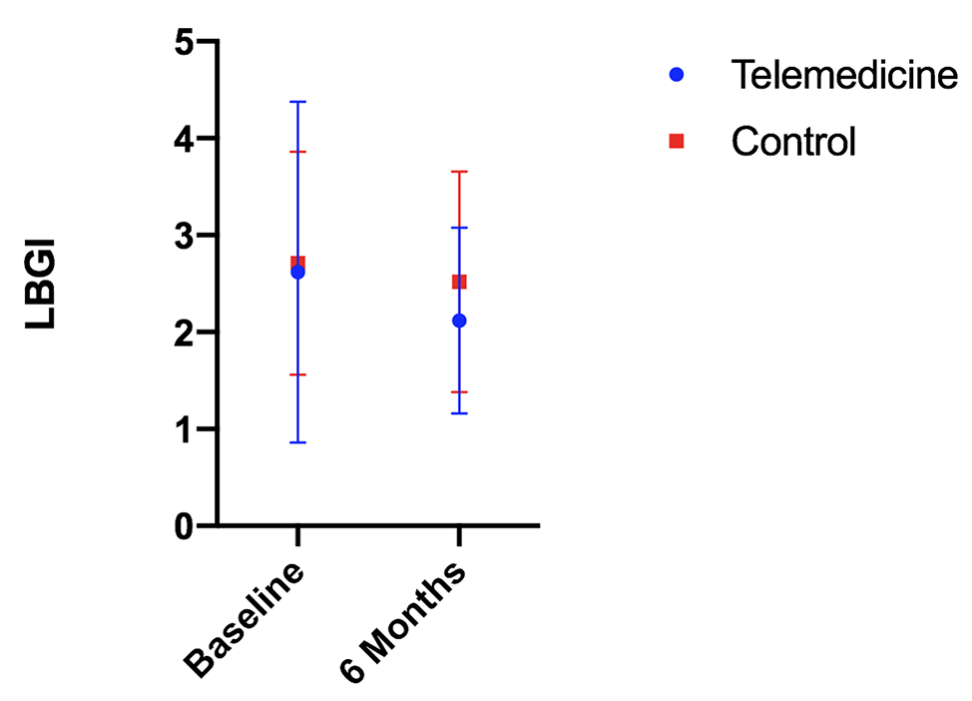
The baseline LBGI for the intervention group and the control group were 2.62(SD 1.76) and 2.71(SD 1.15), respectively, which decreased to 2.12(SD 0.96) and 2.52(SD 1.14) by month 6. LBGI: low blood glucose index

The baseline LBGI values for the intervention group and the control group were 2.62 (SD: 1.76) and 2.71 (SD: 1.15), respectively, which decreased to 2.12 (SD: 0.96) and 2.52 (SD: 1.14) by month 6, respectively. The paired *t* test found a significant difference within the intervention group but not within the control group. The independent-samples *t* test showed a significant difference between the intervention and control groups after 6 months(*P* = 0.04), as shown in Table 2; Fig. 3.

**Fig. 4:**
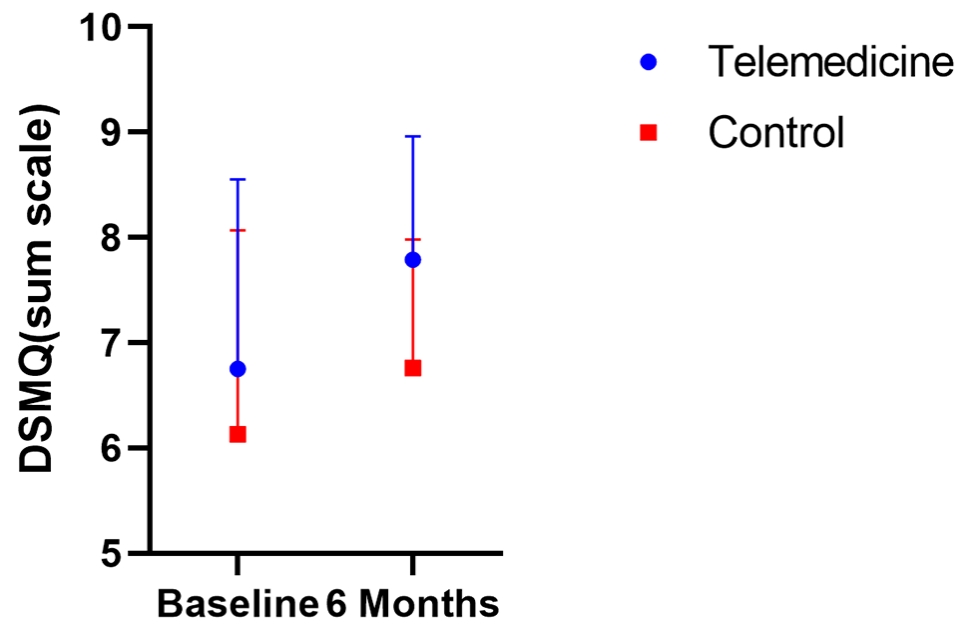
The baseline DSMQ for the intervention group and the control group were 6.75(SD 1.80) and 6.13(SD 1.94), respectively, which decreased to 7.79(SD 1.17) and 6.67(SD 1.22) by month 6. SDMQ: Diabetes Self-Management Questionnaire

The baseline DSMQ results for the intervention group and the control group were 6.75 (SD: 1.80) and 6.13 (SD: 1.94), respectively, which increased to 7.79 (SD: 1.17) and 6.67 (SD: 1.22) by month 6, respectively. As shown in Table 2; Fig. 4, the paired-samples *t* test indicated that the DSMQ values significantly increased within the intervention group after 6 months(*P* < 0.001), although this was not the case within the control group. Additionally, there was a significant difference between the intervention and control groups after 6 months(*P* < 0.001).

## Other outcomes

Overall, there was no difference between the intervention and the control group in terms of BMI, systolic BP, diastolic BP, TC, HDL, and LDL at 6 months. However, the independent-samples *t* test revealed a significant difference in TG between the intervention and control groups after 6 months, as demonstrated in Table 2. Furthermore, there was no difference between the groups in the number of emergency department visits at 6 months (Table 2). Contact rates (app sessions or coaching calls) were significantly higher in the telemedicine group than in the control group, while no differences between study arms were found in face-to-face visits (Table 3).

## Discussion

This study examined the results of a telemedicine system (involving the use of telemedical devices and a coaching app) on the indicators of T2DM control over a 6-month period; the results demonstrated the superiority of the telemedicine system over the traditional approach for improving HbA1c. Additionally, we found a significant reduction in HbA1c levels independent of the BGM testing frequency. As shown in Table 2, the BGM test frequency of subjects in telemedicine group decreased in the last 3 months, but the level of HbA1c still decreased. Furthermore, as the differences in LBGI and DSMQ values between the groups after 6 months were found to be significant, the telemedicine-assisted SMBG improved the risk of hypoglycemia and enhanced diabetes self-management behaviors. Finally, a significant reduction in both TG and the number of visits to specialists after 6 months was observed in the intervention group.

Regardless of the frequency of SMBG by patients, the positive change in HbA1c demonstrates that the telemedicine system facilitates the personalization of SMBG. A structured SMBG strategy may help patients within their daily routines to maintain as normal a BG level as possible by making appropriate food choices (with low/high carbohydrate intake) and lifestyle choices [7]. In addition, the app categorizes the data uploaded by the telemedical devices automatically (high, normal, low), which improves the detection of severe hyperglycemia or hypoglycemia. This increases the understanding of hypoglycemia and helps reduce anxiety regarding the condition [8]. This is consistent with our results: The differences in LBGI between the groups after 6 months were found to be significant; therefore, telemedicine-assisted SMBG improved the risk of hypoglycemia and increased RIR.

Telemedicine-assisted structured SBMG has been shown to help patients by improving metabolic indicators, even if patients don’t monitor blood sugar frequently [24]. We analyze that this is because patients can learn disease related knowledge and self-management skills through the coaching application, which can help them to make decisions and enable users to identify specific situations, even without the guidance and support of doctors. The app can help patients get diet control and achieve physical activity goals. Besides, it helps doctors to monitor patients’ BG, BP, weight and treatment, which may reduce fluctuation of BG [25]. Strengthen self-management behavior of diabetes is the key factor to improve the prognosis of the disease and reduce the risk of diabetes related complications [22, 26]. DSMQ is the preferred tool, which was based on specific diabetes-related questions for analyzing self-reported behavioral problems related to blood glucose control [22]. It is hypothesized that improved self-management leads to improved HbA1c values, which is confirmed by the results presented herein.

This study revealed a significant difference in TG between the intervention and control group after 6 months, which may have been due to patient lifestyle changes. Telemedicine-assisted interventions in lifestyle to change negative attitudes and promote healthy lifestyles include smoking cessation, dietary and exercise prescription, and diabetes education [27]. Furthermore, there was evidence of significance between groups in contact rates (app sessions), indicating that the telemedicine-assisted SBMG is not only a good tool for glycemic control, but also reflects the patient-centered management philosophy of chronic diseases [28].

Telemedicine-assisted SBMG enhances interactions between patients with diabetes and diabetes specialists [29]. Further support for self-decision-making processes in diabetes care may lead to fewer unexpected visits to specialists. This study found that the incidence of hypoglycemia was lower in the telemedicine group than in the control group; this possibly occurred in conjunction with improved compliance with SMBG and medication [30], suggesting that telemedicine can help guide patients to adopt a primary self-care role.

All the participants who were recruited in our study resided in rural areas; they had difficulty accessing hospitals and were potential users of telemedicine. Compared with urban residents, they need telemedicine systems and doctors’ guidance more. However, most previous similar studies selected urban patients [31], the data of rural population is missing. The subjects selected in this study are type 2 diabetes patients in the suburbs of Shanghai, who are not convenient for medical treatment. The BGM used in this study can be directly connected to the app (Huayi Glucose Butler) through Bluetooth. The testing results can be directly transmitted to the app and doctors can provide guidance to patients anytime and anywhere if they need help. These were strengths and unique aspects which distinguish the study from others. However, our research had two limitations. First, the overall follow-up period of 6 months was relatively short. Second, due to the study’s design, we did not have data from patients who failed to attend their appointments or undergo laboratory tests. All of this might have significantly affected the quality of the data.

**Table 3:** Distribution of contracts from 0 to 6 months

	Group	Number of contacts during the first 3 months	Number of contacts during the last 3 months	Overall number of contacts during 6 months	Incidence rate ratios and 95% confidence intervals
All contacts	Telemedicine group	2827	2056	4883	**2.35(1.89–5.20)**
	Control group	1216	862	2078	1.0(RC)
App sessions	Telemedicine group	1292	786	2078	-
	Control group	0	0	0	-
Coaching calls	Telemedicine group	1332	1180	2512	**1.41(0.71–1.56)**
	Control group	1012	764	1776	1.0(RC)
Face-to-face visits	Telemedicine group	203	90	293	0.97(0.61–1.45)
	Control group	204	98	302	1.0(RC)

RC reference class

## Conclusions

Telemedicine-assisted structured SBMG is an important component of modern therapy for T2DM due to its educational and therapeutic role. The system helps physicians and patients to achieve a specific level of glycemic control and prevent hypoglycemia. The use of coaching app (Huayi Glucose Butler) together with telemedicine-assisted SMBG indicated beneficial effects with regard to glycemic control, diabetes self-management, and other metabolic indicators (For example, TG). Currently, the main focus of daily care in most medical systems is medical conditions rather than diabetes self-management education and support. This problem can be addressed by the promotion of telemedicine assisted structured SBMG.

## Data Availability

The datasets used and/or analysed during the current study available from the corresponding author on reasonable request.

## Abbreviations

SMBG: Self-monitoring of blood glucose
T2DM: Type 2 diabetes mellitus
HbA1c: Glycated hemoglobin
LBGI: Low blood glucose index
DSMQ: Diabetes Self-Management Questionnaire
DM: Diabetes mellitus
T1DM: Type 1 diabetes mellitus
App: Applications
FBG: Fasting blood glucose
BMI: Body mass index
BP: Blood pressure
TC: Total cholesterol
TG: Triglycerides
HDL: High density lipoprotein
LDL: Low density lipoprotein
2hBG: 2h-postprandial blood glucose
BGM: Blood glucose meter
OAD: Oral hypoglycemic drugs
RC: Reference class
RIR: Readings in-range
